# Bioactive Pectin-Murta (*Ugni molinae* T.) Seed Extract Films Reinforced with Chitin Fibers

**DOI:** 10.3390/molecules26247477

**Published:** 2021-12-10

**Authors:** Gustavo Cabrera-Barjas, Aleksandra Nesic, Gaston Bravo-Arrepol, Saddys Rodríguez-Llamazares, Oscar Valdés, Aparna Banerjee, Johanna Castaño, Cédric Delattre

**Affiliations:** 1 Unidad de Desarrollo Tecnológico, Parque Industrial Coronel, Universidad de Concepción, Concepción 3349001, Chile; anesic@vin.bg.ac.rs (A.N.); g.bravo@udt.cl (G.B.-A.); 2 Department of Chemical Dynamics and Permanent Education, Vinca Institute of Nuclear Sciences, University of Belgrade, Mike Petrovica-Alasa 12-14, 11000 Belgrade, Serbia; 3 Centro de Investigación de Polímeros Avanzados (CIPA), Edificio Laboratorio CIPA, Avda. Collao 1202, Concepción 4081112, Chile; s.rodriguez@cipachile.cl; 4 Centro de Investigación de Estudios Avanzados del Maule (CIEAM), Vicerrectoría de Investigación y Postgrado, Universidad Católica del Maule, Talca 3460000, Chile; ovaldes@ucm.cl (O.V.); abanerjee@ucm.cl (A.B.); 5 Facultad de Ingeniería y Tecnología, Universidad San Sebastian, Lientur 1457, Concepción 4080871, Chile; Johanna.castano@uss.cl; 6 Institute Universitaire de France (IUF), 1 Rue Descartes, 75005 Paris, France; 7 Institut Pascal, Université Clermont Auvergne, CNRS, Clermont Auvergne INP, 63000 Clermont-Ferrand, France

**Keywords:** pectin, chitin nanofibers, murta seed extract, antimicrobial films, food packaging

## Abstract

This study investigated the biocomposite pectin films enriched with murta (*Ugni molinae* T.) seed polyphenolic extract and reinforced by chitin nanofiber. The structural, morphological, mechanical, barrier, colorimetric, and antioxidant activity of films were evaluated. The obtained data clearly demonstrated that the addition of murta seed extract and the high load of chitin nanofibers (50%) provided more cohesive and dense morphology of films and improved the mechanical resistance and water vapor barrier in comparison to the control pectin film. The antioxidant activity ranged between 71% and 86%, depending on the film formulation and concentration of chitin nanofibers. The presented results highlight the potential use of chitin nanofibers and murta seed extract in the pectin matrix to be applied in functional food coatings and packaging, as a sustainable solution.

## 1. Introduction

Nowadays, the use of materials that cannot be decomposed in nature is one of the leading problems. As most of the non-degradable synthetic materials are used for food packaging, there is a huge need to find sustainable and ecologically safe solutions to protect food from external influences. In that context, biopolymer films have been widely investigated to examine their application as alternative packaging materials. Some of the main advantages of films based on plant biopolymers, compared to films made of synthetic polymers, is that they are edible, biodegradable, and environmentally friendly. They can be used either as coatings, applied to various foods, or as the packaging itself, where they serve as bags or foils for food packaging. The most important features of edible biobased films and coatings are their mechanical and barrier properties, which primarily depend on the biopolymer’s nature, its concentrations, and drying conditions.

In this work, pectin has been chosen as the main biopolymer matrix to produce biobased edible films, due to its high availability in nature (mostly in citrus and apple fruits) and agro-wastes (fruit peels, pomace). Moreover, pectin is a food-safe material that has been commonly used in the food industry for over 40 years as a thickener, gelling, and stabilization additive. Pectin is a complex polysaccharide composed of methoxy esterified α, D-1,4-galacturonic acid units. High methoxy pectins (>50% of esterified groups) are less soluble in water and can form gels at low pH (below 3.5) in the presence of simple sugars. On the other hand, low methoxy pectins (<50% of esterified groups) are easily dissolved in water and have the ability to form gels in the presence of divalent metal ions. Additionally, both types of pectins have great film-forming abilities [1].

However, pectin films do not possess a high-water-vapor barrier and are not biologically active, i.e., they do not possess any antibacterial, antifungal, and antioxidant activity. Additional additives are required to prolong the shelf-life of films, as well as the food products. To date, several different approaches have been used to process pectin-based food packaging films, by the introduction of different plasticizers, antioxidants, and fillers [2,3,4]. Currently, there is a preference to find additives from natural resources instead of the use of synthetic ones, to minimize the negative impact on the environment.

Murta or murtilla (*Ugni molinae* T.), a native plant of the South of Chile, is highly valued as a natural medicine. Fresh fruit consumption and their use for jam, jellies, and liquor preparation have increased during recent years. In the latter cases, industrialization generated seed by-products can be used for value-added product design, e.g., seed bioactive extracts. Moreover, water and methanolic/ethanolic extracts from murta leaves and seeds proved to have a high content of polyphenols and high antioxidant and antimicrobial activity [5,6].

In this work, for the first time, murta seed extract has been used as a natural source of bioactive components to enhance the biological and mechanical properties of pectin films. In addition, to improve the water vapor permeability barrier, chitin nanofibers have been introduced in the system. Namely, due to nanoscale dimensions with a large aspect ratio and high surface area, chitin nanofibers have attracted a great deal of interest as reinforcing nanofillers in the biopolymer matrix [7,8,9]. Hence, the influence of murta seeds in single- and multi-systems combined with different concentrations of chitin nanofibers on the mechanical, water vapor barrier, morphological, and biological properties have been investigated in this paper.

## 2. Results and Discussion

### 2.1. Mechanical and Barrier Properties of Films

Results of tensile strength, elongation at break, and water vapor permeability of pectin-based films are presented in Table 1. The control pectin film (P1) has a moderate tensile strength (18.3 MPa) and plasticity (23.9%). The addition of murta seed extract in the pectin matrix slightly decreased the mechanical stability of pectin films, which was evidenced by lower tensile strength (15.3 MPa). On the other hand, elongation at break increased up to 32.6% due to phenolic compounds present in the extract, that are known to act as plasticizers of biopolymer films [5,10,11]. The incorporation of 10 wt.% of chitin nanofibers did not lead to significant changes in the mechanical properties of pectin-based films, probably because of the non-homogenous distribution of fibers into the polymer matrix. However, the further increase in chitin nanofibers’ concentration (30% and 50%) enhanced the tensile strength in 36.6% (P4) and 83.0% (P5), whereas elongation at break decreased in 23.0% (P4) and 44.5% (P5) in comparison to the control P1 film. This result is probably the outcome of the more homogenous distribution of chitin nanofibers in the pectin matrix and the formation of a denser and more cohesive network. The increased tensile strength and decreased flexibility of biopolymer films with the addition of chitin nanofibers in starch [7] and carboxymethyl cellulose matrix [12] have also been reported in the literature.

WVP of control pectin film was 4.37 × 10^−11^ g/m s Pa, which is one magnitude better than for data obtained in the literature for pectin/glycerol [13], pectin/PEG films [14], and pectin/polyglycerol films [15]. The reason for the better WVP barrier of pectin film obtained in this work might be based on the different sources and monosaccharide composition of pectin, the different degrees of methylation of pectins that are used, as well as the different types and concentrations of plasticizers. The water vapor permeability of pectin film slightly decreased with the addition of murta seed extract (by 6.85%), in comparison to the control pectin film P1. Similar findings were found in the literature, where the addition of extract from murta leaves into carboxymethylated cellulose [16] and tuna fish gelatin [17] led to a decrease in WVP. Further, the addition of chitin nanofibers into pectin and pectin/murta seed extract films resulted in an enhanced water vapor barrier of films. This result is expected, since it is known that fibers with high aspect ratios enhance the barrier property due to the formation of tortuous paths once they are properly and sufficiently dispersed in the polymer matrix [18]. The best WVP barrier was achieved for sample P5, which contains 50% of chitin fibers, reaching a value of 1.12 10^−11^ g/m s Pa, which is better in comparison to the control pectin film (P1).

TS: tensile strain; Eb: elongation at break; WVP: water vapor permeability. According to Tukey’s test, the different letters mean a significant difference (*p* < 0.05).

### 2.2. Color Analysis

**Figure 1:**
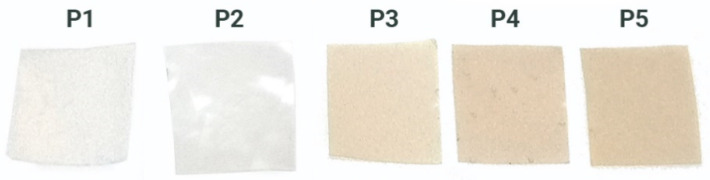
Visual appearance of pectin-based films.

The color parameters of pectin-based films are presented in Table 2, and the visual appearance of films is presented in Figure 1. It was detected that the pectin films have high lightness according to the L* value. The addition of murta seed extract and chitin fibers induced a slight decrease in the L* value, indicating less bright films. The a* value was positive for all tested samples, indicating a reddish color of the film, which was more pronounced within a higher concentration of chitin fibers in the film. The b* value was positive for all samples, indicating that films contain more yellow than blue tones, and this effect was also more pronounced with the increased concentration of chitin fibers in the film. The ΔE* is an indicator of the color change between the samples, and in this case, the color change of pectin-biocomposite films was compared to the control blank. In all cases, ΔE* values were higher than 3.5, confirming that the color change can be visually perceived [19]. Moreover, with an increase of fibers in pectin-based films, the color changes in films were more visible, which was confirmed visually and by ΔE* values.

### 2.3. Thermogravimetric Analysis

**Figure 2:**
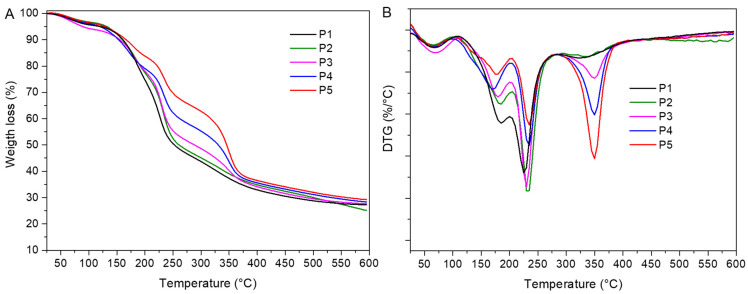
(**A**) TGA and (**B**) DTG diagrams of pectin-based films.

The TG-DTG curves of the pectin-based films are presented in Figure 2 and results are summarized in Table 3. In all films, a first thermal effect ranging from 25 to 103 °C, and peaking from 63 to 77 °C, was associated with the loss of bound water from films [14]. Pectin-glycerol film (P1) showed a second thermal effect ranging from 109 to 204 °C, and T_peak_ 184 °C, which belongs to the weight loss of unbound water and glycerol (19.8%) from the films. The inclusion of murta seed extract into the pectin/glycerol matrix (P2) did not change its thermal stability, which was evidenced by the position of maximum degradation temperature (T_peak_) and the temperature at which degradation starts (T_onset_).

In all films, a third thermal degradation step occurred from 205 to 292 °C, with a maximum decomposition rate at T_peak_ 230–235 °C, and associated weight loss ranging from 30.1% (P1) to 17.5% (P5), depending on film composition. Lower weight losses were observed at higher content of chitin nanofiber in films. In this stage, pectin hydrolysis, ring dehydration, and decarboxylation occurred [15,20].

The pectin/murta seeds/chitin nanofibers films (P3–P5) showed the same thermal stages as P1 and P2 films, with one additional degradation step in the range between 288 and 388 °C, peaking at T_peak_ 350 °C, which is related to the thermal degradation of chitin nanofibers [21]. During this process, chitin chains degraded in a complex manner due to simultaneous ring dehydration, glycosidic linkage hydrolysis, and the thermal decomposition of acetylated and deacetylated units, respectively. As a consequence, the chitin crystalline structure and packaging were lost. The presence of chitin nanofibers in the polymer matrix did not induce significant changes in T_onset_ and T_peak_ values compared to the degradation pattern of the control film P1. Hence, the thermal stability of pectin films was maintained with the addition of a bioactive component and a nanofiller.

### 2.4. FTIR/ATR Analysis

**Figure 3:**
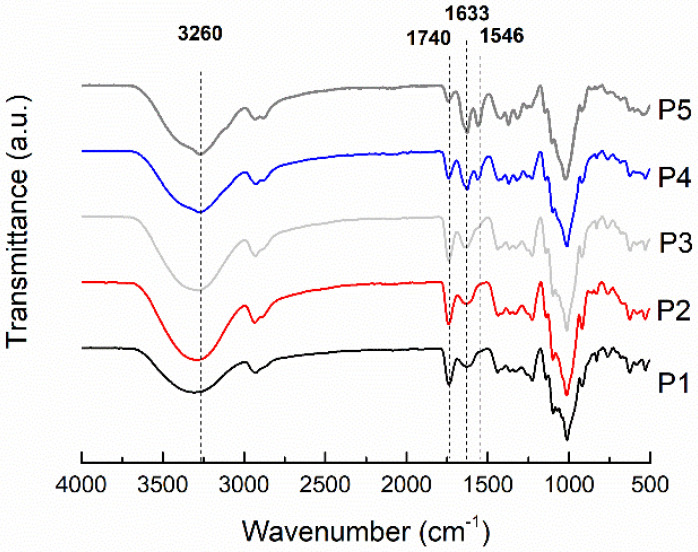
FTIR/ATR spectra of control pectin film and pectin-based biocomposite film.

FTIR spectra of representative pectin biocomposite films are shown in Figure 3. The FTIR spectrum of the pectin-based film shows bands associated with the polysaccharide structure: around 3300 (stretching vibrations of -OH groups), 2930 (stretching vibrations of -CH_2_, -CH_3_ groups), and 1014 cm^−1^ (stretching vibrations of C-O-C) [2,3]. The broad peak at 1630 cm^−1^ is attributed to C=O stretching of the non-esterified uronic carboxyl groups (from galacturonic acid units) of pectin (P1 and P2). The murta seed extract addition (P3–P5) did not change the FTIR spectra of the biocomposites, and all of them were overlapped by the one from other majority components. The incorporation of chitin fibers into the pectin matrix caused the appearance of the peaks at 1560 cm^−1^ (the -NH bending and -CN stretching vibrations of amide II) and 3260 cm^−1^ (stretching vibrations of the -NH groups), associated with β-chitin fibers [1]. As the loading of chitin fibers increased from 10% to 50%, the intensity of both peaks increased in the spectra of the pectin biocomposite films (P3, P4, and P5). In addition, the band associated with the deformation, -NH, and stretching, -CN, vibrations of chitin (amide II) was shifted to higher wavenumbers, from 1546 to 1560 cm^−1^. This suggests that the regular N-H...N and O-H...N hydrogen bonding interactions in chitin nanofibers are disrupted in pectin biocomposite films. The shift to higher wavenumbers has been reported for poly(ethylene oxide) (PEO)/chitin nanocomposite films, where the interactions through hydrogen bonds between the NH groups of the chitin fiber surface and the oxygen of the PEO ether groups (weak interactions) predominate over chitin’s interactions [6]. The formation of an interpolymeric complex of the chitin fiber with the pectin matrix can be concluded from the shift of the amide II peak to higher frequencies.

### 2.5. SEM Analysis

**Figure 4:**
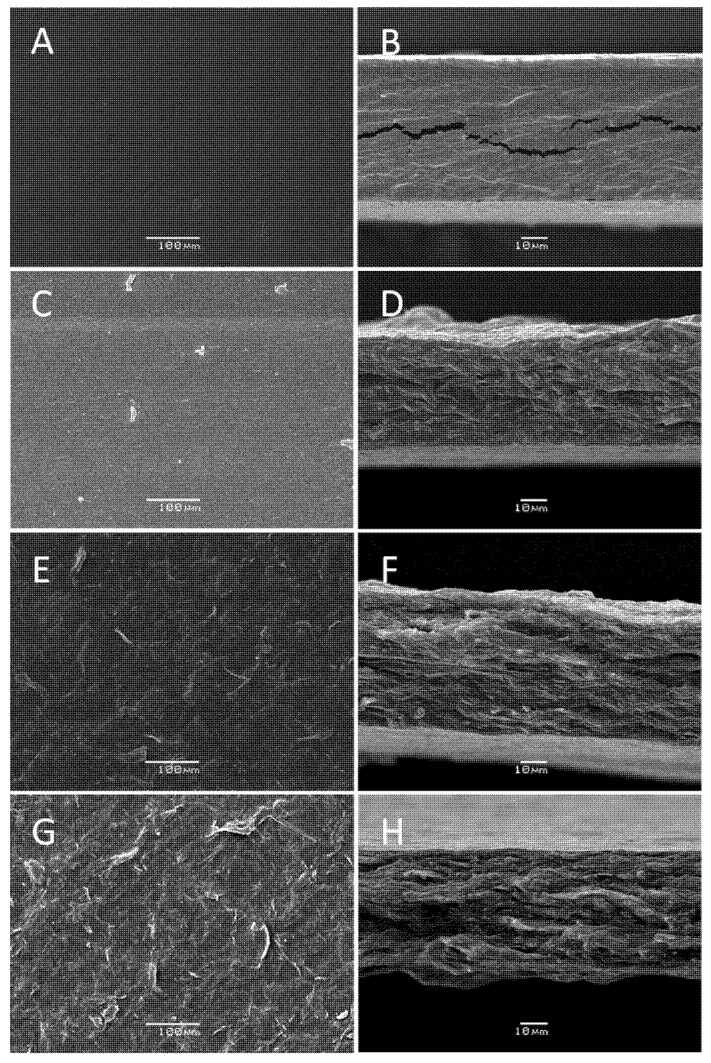
SEM analysis of pectin and pectin biocomposite films with different compositions showing the surface and the cross-section view: P1 (**A**,**B**), P3 (**C**,**D**), P4 (**E**,**F**), and P5 (**G**,**H**).

The biocomposite film morphological studies were carried out by SEM, and results are shown in Figure 4. The control pectin/glycerol film (Figure 4A,B) shows a smooth surface and a compact structure. Once the chitin nanofibers and murta extract were added to the pectin film, the morphology of the films changed drastically (Figure 4C–H). A rising rougher and fibrous surface at the surface and cross-section of the films was observed with the chitin nanofiber content increase. The obtained morphology of these films resembles the one previously obtained with pure chitin nanofiber films [21]. This indicates that a well-blended and compatible biocomposite film was formed. It is suggested that the electrostatic interactions between chitin –NH_3_^+^ groups on the surface of the nanofibers and pectin –COO^−^ groups lead to the formation of polyelectrolyte complex [22], thus contributing to the film stability. Additionally, hydrogen bonding among pectin chains and pectin-chitin fibers is expected.

### 2.6. Antioxidant Activity of Films

The results of DPPH free radical scavenging of pectin-based films were presented in Table 1. Interestingly, there were significant (*p* < 0.05) statistical differences among all treatments. Control pectin-glycerol film did not have antioxidant activity, as was expected, because it does not contain polyphenolic extract. The highest antioxidant activity (86%) was in the sample P2, which contains pectin, glycerol, and murta seed extract. It can be observed that the higher the content of chitin nanofibers in film formulation, the lower the antioxidant activity. The release of polyphenolic extract in ethanol solution was low in the first 24 h, and it was modulated by chitin nanofibers content. This result was expected since SEM analysis confirmed that a more cohesive and fibrous dense network was formed with the addition of chitin nanofibers, which probably prevents the fast release of extract from the film. These results agree with other polysaccharide-based films loaded with natural antioxidant extracts showing a sustained release of the polyphenolic compounds [23,24].

## 3. Materials and Methods

### 3.1. Materials

Citrus pectin (galacturonic acid content 74.3%, degree of methylation 63% mol, Mw 615 kDa) and glycerol were purchased from Sigma-Aldrich and used without further purification. According to the procedure reported in our previous paper, an aqueous dispersion of chitin nanofibers was produced in the lab [18]. The murta (*Ugni molinae* T.) seed polyphenolic extract was obtained and characterized according to our previous work [5]. The aqueous solution of murta (*Ugni molinae* T.) seed extract was obtained by dissolving the lyophilized extract in distilled water at a concentration of 30 wt.%, and further used in experiments in that form.

### 3.2. Film Preparation

Pectin solution was prepared by dissolving an appropriate amount of biopolymer in distilled water and stirring at 70 °C until a complete homogenous solution was obtained. Next, the glycerol was added, followed by the chitin nanofibril aqueous dispersion, and then the aqueous solution of the murta seed extract. The ratio between pectin and chitin nanofibers was varied from 100% to 50%, but the total concentration of pectin and chitin nanofibers in all solutions was 1 wt.%. The glycerol and murta seed extract content was kept constant in all film formulations and calculated by the total weight of biopolymer concentration in solution. The film mixtures were agitated until homogeneity, poured (30 mL) into plastic Petri dishes (diameter 10 cm), and dried in an oven at 60 °C for 24 h. The codes and film formulations are listed in Table 4.

### 3.3. Mechanical Test

Film samples (five centimeters long and half a centimeter wide) were exposed to a tensile test using a smarTens 005 Universal Tensile Testing Machine, by Karg Industrietechnik. The test strips were clamped into place and pulled apart by the machine until they broke. The cross-spread was 2 mm/min. The presented results are the average values of 6 measurements.

### 3.4. Color Measurements

The colorimetric analysis of obtained films was scanned by the Biobase BCM-200 colorimeter (Biobase Meihua Co., Jinan, China). Measurements were performed using the CIE L* a* b* system, where the parameter L* presents the brightness level (from 0—pure black to 100—diffuse white), a* extends from green (negative a*) and red (positive a*), and b* from blue (negative b*) to yellow (positive b*). The total color difference is presented by the ΔE* parameter, which is calculated according to Equation (1):

(1) $${\Delta E}^{*}=\sqrt{\left(L_{0}^{*}-L^{*}\right)+{\left(a_{0}^{*}-a^{*}\right)}^{2}+{\left(b_{0}^{*}-b^{*}\right)}^{2}}$$

where $L_{0}^{*}$, $a_{0}^{*}$, and $b_{0}^{*}$ are the values of the film before immersion into different pH solutions.

### 3.5. Water Vapor Permeability

The water vapor permeability (*WVP*) of the films was performed gravimetrically following the standard method of ASTM E96-95. The films were sealed on the top of a permeability cup filled with silica gel. All cups were stored in an environmental chamber set at a temperature of 25 °C and an RH of 60%. The cups were weighed every day until the equilibrium was reached. The water vapor permeability of films was calculated according to the following equation, and water vapor transmission rate (*WVTR*) values, expressed in g/(h m), were determined from the linear plot of weight change vs. time, following Equation (2):

(2) $$WVTR=\frac{\Delta G}{tA}$$

where water vapor permeability was calculated according to Equation (3):

(3) $$WVP=\frac{\Delta GL}{tA\Delta P}$$

where Δ*G/t* (g/h) was the slope in the linear plot of weight vs. time, *L* (m) was the thickness of the film, and Δ*P* was the water pressure difference between both sides of the film (Pa). The data were presented as the mean of three measurements for each sample.

### 3.6. Thermogravimetric Analysis

Thermogravimetric analysis was performed on a NETZSCH TG 209 F3 Tarsus^®^ thermal analyzer (MB & Cia, Selb, Germany). The samples were heated from 30 to 600 °C at a heating rate of 10 °C/min, under a nitrogen atmosphere with a nominal gas flow rate of 30 mL/min. For each composition, the thermogravimetric tests were performed in duplicate.

### 3.7. Fourier Transform Infrared Spectroscopy

FTIR/ATR analysis was performed on a Jasco FT/IR 400 spectrometer. The spectra were scanned in the range of 4000–480 cm^−1^ at a resolution of 4 cm^−1^.

### 3.8. SEM Analysis

The obtained films were scanned by an ETEC auto scan Model U-1 scanning electron microscope (University of Massachusetts, Worcester, MA, USA) to evaluate the morphological structure of samples. Before the scanning, the samples were sputtered with a gold layer for 3 min, using an Edwards S150 sputter coater (BOC Edwards, São Paulo, Brazil), before analysis.

### 3.9. Antioxidant Activity of Films

DPPH antioxidant activity was determined according to the following procedure: Film samples with a size of 1.5 × 1.5 cm were mixed with 1 mL of ethanol. The mixture was vigorously vortexed for another 3 min and stirred in a thermoshaker at 25 °C for 24 h. Afterward, it was vigorously vortexed for another 3 min and centrifuged at 3000 rpm for 10 min. An aliquot of ethanol extract (100 µL) was mixed with 1 mL of 0.2 nM DPPH in ethanol. The mixture was vigorously vortexed for 1 min and allowed to stand at room temperature in the dark for 30 min. The absorbance was measured at 517 nm using a UV spectrometer. Results are presented as DPPH inhibition percentage.

### 3.10. Statistical Analysis

The antioxidant activity experimental data were subjected to statistical analysis using the SPSS^®^ v.11.0 Software (IBM, Chicago, IL, USA), and ANOVA was performed. The Tukey’s test was used to compare differences among means at the *p* < 0.05 level.

## 4. Conclusions

Biocomposite pectin films loaded with murta seed extract and chitin nanofibers were successfully prepared by using the solution casting method. Good compatibility among all components in the films was demonstrated by FTIR. Besides, the thermal stability of pectin-based films was not significantly changed after nanofiber and extract addition. It was determined that chitin nanofiber content modulates the films’ ultimate physicochemical, mechanical, and barrier properties, improving them. SEM analysis demonstrated that a high load of chitin nanofibers in the pectin matrix led to a more dense and fibrous morphology of the films, which prevented high release of murta seed extract from films, and as a consequence, decreased the antioxidant activity from 86% (control P2 film) to 71% (P5 film, with 50 wt.% of chitin nanofibers). These findings suggest the possibility of using chitin nanofibers for the synthesis of polysaccharide films, having antioxidant-sustained release properties. On the other hand, P5 film formulation exhibited the highest tensile strength (33.5 MPa) and the lowest water vapor barrier (1.12 × 10^−11^ g/m s Pa). Overall, these results represent a significant aspect of the successful use of chitin nanofibers and murta seed as a source of antioxidant polyphenols to improve the properties of pectin-based films for food packaging applications.

## Figures and Tables

**Table 1:** Mechanical parameters, WVP, and antioxidant activity of pectin-based biocomposite films.

Sample	TS (MPa)	Eb (%)	WVP × 10^11^ (g/m s Pa)	DPPH (Inhibition %)
P1	18.3 ± 1.2	23.9 ± 2.1	4.37 ± 0.3	0 a
P2	15.6 ± 0.9	31.7 ± 2.5	4.07 ± 0.2	86 ± 2 e
P3	14.4 ± 0.8	32.4 ± 2.2	3.74 ± 0.2	81 ± 2 d
P4	25.0 ± 1.5	18.4 ± 1.5	2.41 ± 0.2	77 ± 2 c
P5	33.5 ± 2.2	13.3 ± 1.1	1.12 ± 0.05	71 ± 2 b

TS: tensile strain; Eb: elongation at break; WVP: water vapor permeability. According to Tukey’s test, the different letters mean a significant difference (*p* < 0.05).

**Table 2:** Color analysis of pectin-based biocomposite films.

Sample	L*	a*	b*	ΔE*
P1	90.553	0.611	2.409	4.482
P2	90.366	3.537	5.018	7.471
P3	83.895	3.912	7.297	12.623
P4	81.121	3.678	8.166	15.105
P5	77.851	4.226	13.037	20.853

**Table 3:** Thermogravimetric properties of pectin-based films.

Sample Code	Temperature (°C)			Weight Loss (%)
	T_ONSET_	T_PEAK_	T_END_	
P1	25	63	95	3.1
	109	184	204	19.8
	205	232	292	30.1
P2	25	66	102	3.5
	108	186	204	20.4
	205	232	279	28.6
P3	25	70	103	6
	104	179	200	16
	204	230	279	26.1
	288	350	387	16
P4	25	66	100	3.9
	102	170	201	17.1
	204	235	279	20.6
	288	350	388	21.5
P5	25	67	99	3.5
	100	175	200	13.4
	204	235	279	17.9
	288	350	387	28.2

**Table 4:** Codes and film formulations.

Sample Code	Pectin/Chitin Nanofibril Ratio (wt.%)	Glycerol (wt.%)	Murta Seed Extract (wt.%)
P1	100/0	30	0
P2	100/0	30	10
P3	90/10	30	10
P4	70/30	30	10
P5	50/50	30	10

## Data Availability

Not Applicable.

## Sample Availability

Not available.
